# Multiple insecticide resistance mechanisms in primary dengue vector, *Aedes aegypti* (Linn.) from dengue endemic districts of sub-Himalayan West Bengal, India

**DOI:** 10.1371/journal.pone.0203207

**Published:** 2018-09-10

**Authors:** Minu Bharati, Dhiraj Saha

**Affiliations:** Insect Biochemistry and Molecular Biology Laboratory, Department of Zoology, University of North Bengal, Raja Ramohunpur, P.O. North Bengal University, Siliguri, District – Darjeeling, West Bengal, India; National Taiwan Ocean University, TAIWAN

## Abstract

**Background:**

Mosquitoes belonging to genus *Aedes* are the prime vectors of several arboviral diseases such as Dengue, Zika and Chikungunya worldwide. Every year numerous cases of dengue infections occur throughout the world, proper control of which depends on efficient vector control. However the onset of insecticide resistance has resulted in failure of vector control approaches.

**Principal findings:**

This study was carried out to unveil the degree of prevailing insecticide resistance along with its underlying mechanisms among the primary dengue vector in dengue endemic districts of West Bengal, India through standard WHO protocol. It was observed that, the majority of the tested populations were found to possess resistance to more than one insecticide. In adult bioassay, the toxicity levels of the six tested insecticides was found to decrease in the following order: deltamethrin > lambdacyhalothrin > malathion > propoxur > permethrin > DDT. In larval bioassay, one of the tested populations was found to possess moderate resistance against temephos, mortality percentage 92.5% and 79.8% for WHO (0.0200 ppm) and National Vector Borne disease Programme, India recommended dose (0.0125 ppm) respectively. Carboxylesterases were found to be involved in conferring resistance as revealed in synergistic and quantitative assay against temephos in North Dinajpur (NDP) population and malathion in Alipurduar (APD) and Darjeeling (DAR) populations. Similar correlations were also observed in the majority of the tested populations between reduced susceptibilities against pyrethroid insecticides and Cytochrome P_450_s activity.

**Conclusion:**

Efficient disease management in this region can only be achieved through proper integrated vector management along with tools to minimize insecticide resistance. This study may help the concerned authorities in the formulation of an effective vector control strategy throughout this region incorporating the knowledge gained through this study.

## Introduction

Mosquitoes transmit diseases of public health importance such as dengue, chikungunya, malaria, filariasis *etc*, thus presenting a threat to human health. *Aedes* mosquitoes namely, *Ae*. *aegypti* and *Ae*. *albopictus*, key vectors of dengue virus (DENV) and chikungunya virus (CHIKV) [1–2] have recently invaded different geographical regions throughout the world [3]. Annually, dengue, chikungunya and yellow fever collectively infect around 75 million people, with 25,000 deaths [1]. Recently, a new dengue serotype has appeared in the Asian continent that follows the sylvatic cycle unlike the other four serotypes which follow the human cycle [4–5]. Emergence and spread of such new serotypes may enhance the severity of the disease.

Since last few years, annually more than one lakh cases of dengue infections occur in India [6] resulting in substantial rates of mortality and morbidity. In the state of West Bengal, 10,697 people were infected with dengue in 2017 with 19 deaths [6]. This state provides an ideal *Aedes* mosquito breeding environment owing to the presence of large vegetation cover and high rainfall [7].

In absence of specific medications against dengue the sole method of disease prevention relies on control of vector mosquitoes. The prevention and control of dengue in India is followed through integrated vector management which includes entomological surveillance; following source reduction, use of larvicides and larvivouros fish, environment management as anti larval measures; and following regular anti adult measures through either indoor residual spray by 2% pyrethrum extract or fogging by 5% malathion during disease outbreaks [6]. Additionally, some commercially available mosquito control/repellant tools are also widely used in India by the general public (for personal protection) which contain compounds mainly belonging to pyrethroid group of insecticides.

Due to indiscriminate use of insecticides, mosquitoes have evolved strategies to resist the planned actions of insecticides in their bodies, this phenomenon is known as insecticide resistance [8]. Mosquitoes have developed insecticide resistance both as a direct effect of insecticides targeted on them as well as an indirect exposure of insecticide sprayed on agricultural field [7,9–10]. Insecticide resistance is the major obstacle nowadays in efficient vector/pest control approaches. Altered susceptibilities of *Aedes* species to insecticides could be either governed by metabolic detoxification through enzyme systems present in the body or through altered target site in field populations. Over expression or gene amplification of enzyme families/classes, Carboxylesterases (CCEs), Glutathione S-transferases (GSTs) and Cytochrome P_450_s (CYP_450_s) or Mixed Function Oxidases (MFOs) have been shown to confer insecticide resistance in many populations of insecticide resistant *Aedes* mosquito population worldwide [1,11]. Moreover, target site alteration either as a result of point mutations in voltage gated sodium channel gene or an insensitive AchE mechanisms have been identified in vector mosquitoes [1,11]. Knockdown resistance (kdr) mutations, *i*.*e*. mutation in voltage gated sodium channel are widespread in *Aedes* population and have been shown to provide selective advantage over pyrethroid and organochlorine insecticide pressure in many populations of *Aedes aegypti* [1,12].

Identification of prevailing level of insecticide resistance along with its underlying mechanisms have important implications for vector control. The findings of this study may be helpful in designing efficient integrated vector control strategies along with tools to combat insecticide resistance during intense disease outbreaks.

## Materials and methods

### Selection of sampling districts and mosquito collection

**Fig 1:**
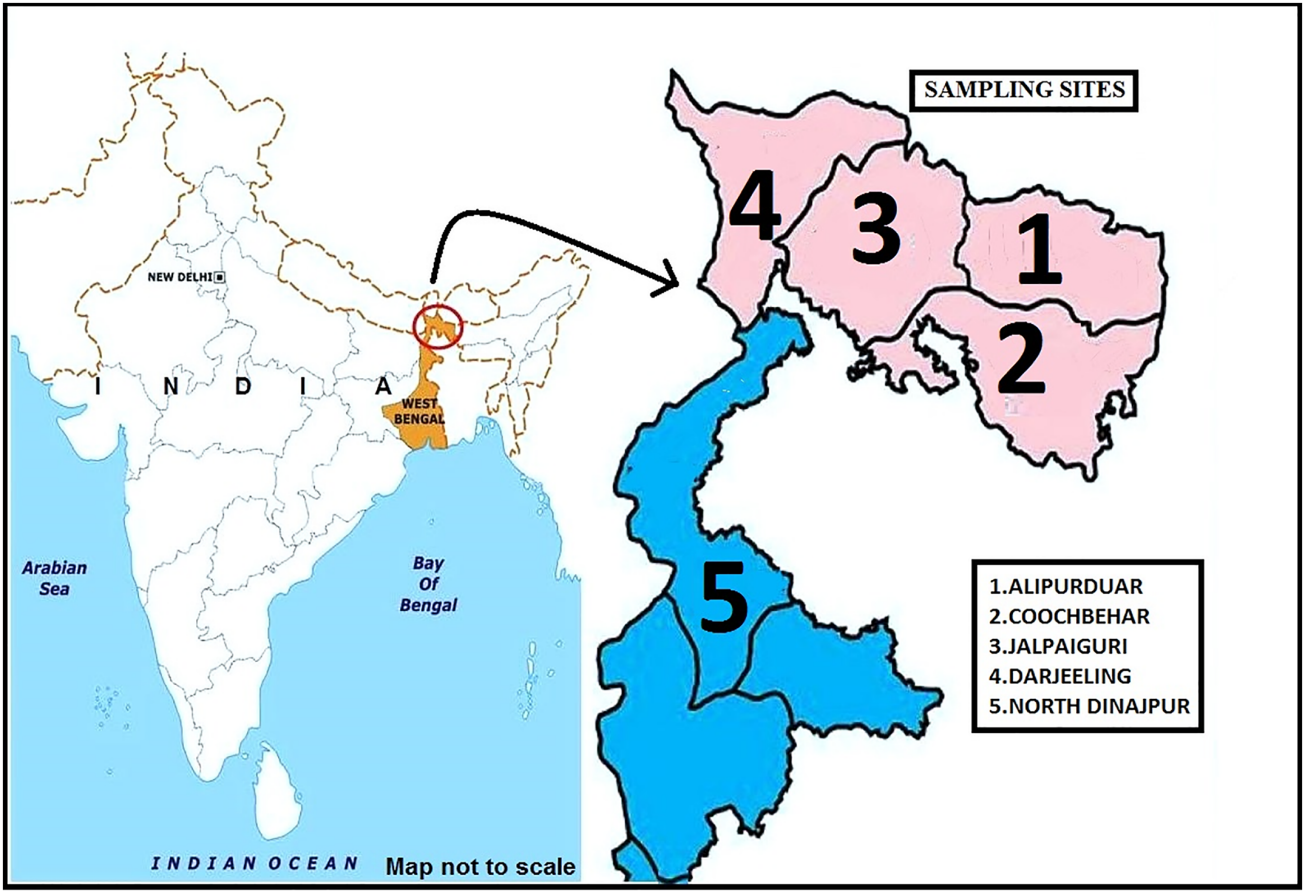
Map of the sampling sites.

Five different sampling districts were selected in northern part of West Bengal, namely, Alipurduar, Jalpaiguri, Darjeeling, Coochbehar and North Dinajpur. The relevant biotic and abiotic factors of the sampling sites are provided in Table 1. The selected sampling sites (Fig 1) were screened for the larva and pupa of *Aedes* mosquitoes. Mosquito larvae/pupae were collected from different wild habitats only such as discarded automobile tyres, earthen pots, artificial containers, water holding tanks, discarded buckets, aloevera plantations, tree holes, pots *etc*. The larvae initially identified as *Aedes* were collected and transferred to plastic containers and brought to the laboratory. The sampling was done during March to November 2017 and March 2018 to April 2018, pre-monsoon, monsoon and post-monsoon seasons and the details of total collection (sampling site and season wise) is provided in Table 1. Since all the sampling was done from private land, prior permission was taken from the land owner for mosquito collection.

**Table 1:** Details of the sampling sites.

Districts	Population name	Geographical coordinates	Total numbers of mosquito (larvae and pupae) sampled	*Mosquito* *G*eneration used in Experiments	Disease endemicity	Last season of dengue outbreak	Total infection in 2016
Alipurduar	APD	26.69° N 89.47° E	2018: Pr M-1067 2017: Pr M-1258 M-907 Po M-1103	F1	Dengue, Malaria, JE	2017	16
Coochbehar	COB	26.34° N 89.46° E	2018: Pr M-965 Pr M-1141 M-701 Po M-922	F1	Dengue, Malaria, JE, Filariasis	2017	37
Jalpaiguri	JPG	26.52° N 88.73° E	2018: Pr M-694 Pr M-967M-1231 Po M-709	F1	Dengue, Malaria, JE, AES	2017	168
Darjeeling	DAR	26.71° N 88.43° E	2018: Pr M- 1165 Pr M-1032M-971 Po M-1121	F1	Dengue, Malaria, JE, AES	2017	165
North Dinajpur	NDP	26.27° N 88.20° E	Pr M- 754 Pr M- 1948M-852 Po M-768	F1	Dengue, Malaria, JE, AES	2017	87
Susceptible population	SP	--	--	F10	--	--	

JE: Japanese Encephalitis, AES: Acute Encephalitis syndrome, Pr M: Pre-monsoon, M: Monsoon,

### Selection and rearing of susceptible and field caught population of mosquitoes

In the laboratory, the larvae were identified upto subspecies level following standard identification keys [13–14]. All the collected mosquitoes were identified to be *Aedes aegypti aegypti*. The field collected larvae (F_0_) were then reared at temperature 25±2°C and 70–80% relative humidity. The rearing was done based on the standard method [7] for successive generations. The larvae were reared to F_1_ generation upto adults to ensure the homogeneity of the field collected populations. The emerged adults were cross checked with adult identification keys [14]. The F1 larvae and adults were used for bioassays and detoxifying enzyme activity studies. To setup a susceptible laboratory culture, mosquito samples were collected randomly from five organically managed areas with lowest insecticide exposure possibilities. The mosquito colonies after collection were reared to F1 generation and were subsequently tested for insecticide susceptibility bioassays. The mosquito population that was recorded to possess the lowest level of resistance (collected from the Medicinal garden of North Bengal University campus, Siliguri, India) was chosen from the rest to be reared for ten additional generations without any exposure to insecticides in the laboratory maintaining the same physical factors as mentioned earlier and provided with anaesthetised rat as a source of blood for the females in each generation to be used as the laboratory reared control/ susceptible population (SP).

### Insecticide

Temephos solution (156.25 mg/L) and insecticide impregnated papers 4% DDT, 0.05% deltamethrin, 0.05% lambdacyhalothrin, 0.75% permethrin, 5% malathion and 0.1% Propoxur were purchased from Vector control unit, Universiti sains Malaysia.

### WHO bioassay

To assess the susceptibility status of adult mosquitoes, thirty (30) 2–3 days non blood-fed adults were exposed to insecticide impregnated papers with WHO recommended diagnostic dose of insecticide (4% DDT, 0.05% deltamethrin and 0.05% lambdacyhalothrin, 0.75% permethrin, 5% malathion and, 0.1% Propoxur) placed in tubes for 1 hour [15]. After one hour, the mosquitoes were transferred to retention tube containing cotton balls soaked in 10% glucose solution. Mortality percentages were recorded 24 hours post-exposure. For control, mosquitoes were place in tubes containing papers impregnated with silicone oil and acetone. For synthetic pyrethroids and organochlorine insecticides, number of knocked down mosquitoes were counted for every ten minutes, to determine the knockdown time, *i*.*e*. KDT_50_ and KDT_95_.

To assess the susceptibility of *Ae*. *aegypti* larvae against temephos standard WHO guidelines were followed [16]. Thirty (30) late third instar or early fourth instar larvae of each population were exposed to test vials containing two different discriminating doses: 1. WHO recommended dose (0.0200 mg/L) and 2. India government recommended dose (0.0125 mg/L) of temephos in water. One set of control (using solvent instead of insecticide solution) was also set under laboratory conditions. Mortality percentage was recorded post 24 hours of temephos exposure. Larvae were considered dead or moribund if they failed to evoke any response when touched [16].

For the determination of resistance ratio, *i*.*e*. RR50 of temephos, standard methodology was followed [7]. Both adult and larval assays were performed in triplicates and the mortality percentages were taken as the average of the three assays.

### Synergism tests

Synergism tests were conducted using the field populations to evaluate the effectiveness of synergists on detoxification of insecticides. Piperonyl butoxide (PBO) (90%, Sigma from Sigma-Aldrich, Singapore), a CYP_450_s inhibitor and triphenyl phosphate (TPP) (99%, from Sigma-Aldrich, Singapore), a CCE inhibitor were used. The sub-lethal doses of both the synergists *i*.*e*. 4% and 10% for PBO and TPP respectively were used in synergism tests. The protocol for the synergism tests were similar to the larval bioassays described above, except that the insecticide was mixed with synergist prior to the test. For adult bioassays, each population was exposed to synergist for one hour prior to insecticide exposure. Diagnostic tests in WHO bioassays section (exposure to insecticide only) served as positive control while bioassays without insecticide were used as negative control.

### Insecticide detoxifying enzymes’ activity

Single adult *Ae*. *aegypti* were homogenized in 100 μL of 0.1M sodium phosphate buffer (pH 7.2) with a teflon micro-pestle in a 1.5 mL centrifuge tube and the whole solution was made 200 μL with 0.1 sodium phosphate buffer. The homogenate was centrifuged at 12,000 rpm (revolutions per minute) for 15 minutes in a centrifuge (Sigma 3K30, Sigma,U.K.) [7]. The supernatant was stored at -20°C and was used within 3–4 days as enzyme source for detoxifying enzyme activity assays. For each biochemical test, a minimum of thirty individuals were assayed. A duplicate set was also run for each enzyme assay. In this study, a single substrate for each enzyme group (two for CCEs) has been used for assessing the enzyme activity levels. Though, an enzyme group may have many substrates, yet the substrates used are identical to substrates used in standard protocols [17].

#### Non-specific esterase (carboxylesterase) assay

The activity of carboxylesterases (CCEs) hydrolyzing α- and β- naphthyl acetate as substrate were assayed according to standard WHO guidelines[17] with minor modifications for using in microplate [7].

#### CYP_450_ assay

The activity of CYP_450_ was also measured according to standard WHO guidelines [17] using 3,3',5,5'-Tetramethyl benzidine (TMBZ) as a substrate and H_2_O_2_ as the peroxidising agent. The total CYP_450_ was expressed as CYP_450_ equivalent units (EUs) in mg protein.

#### Glutathione S-transferase (GST) assay

GST activity was assessed following the WHO protocol [17] using CDNB/GSH as the working solution in wells of microtitre plate.

#### Total protein content

Total protein of each individual of *Ae*. *aegypti* was determined according to standard WHO guidelines [17] to cancel out any size differences among individuals and for the correct expression of enzyme activity.

### Calculation

In the insecticide susceptibility bioassays, no calculation of corrected mortality was needed because control mortalities were below 5%. The population with mortality percentages when > 98 is said to be susceptible, 80–97 is assessed as resistance not confirmed (= unconfirmed resistance = incipient status) and <80 as resistant [15–16]. LC_50_ was estimated at 95% confidence interval by putting log dose against probit in SPSS 16.0 software and the obtained linear regression coefficient (r^2^) was used to assess the linearity of the data set. Resistance ratio 50 *i*.*e*. RR50, which is an indirect measurement of insecticide resistance development was also determined as the LC_50_ of sampling site divided by the LC_50_ of the SP Similarly the knockdown time for 50% (KDT_50_) and 95% (KDT_95_) of tested mosquitoes were calculated using probit analysis.

## Results

### WHO bioassays and synergistic tests

**Fig 2:**
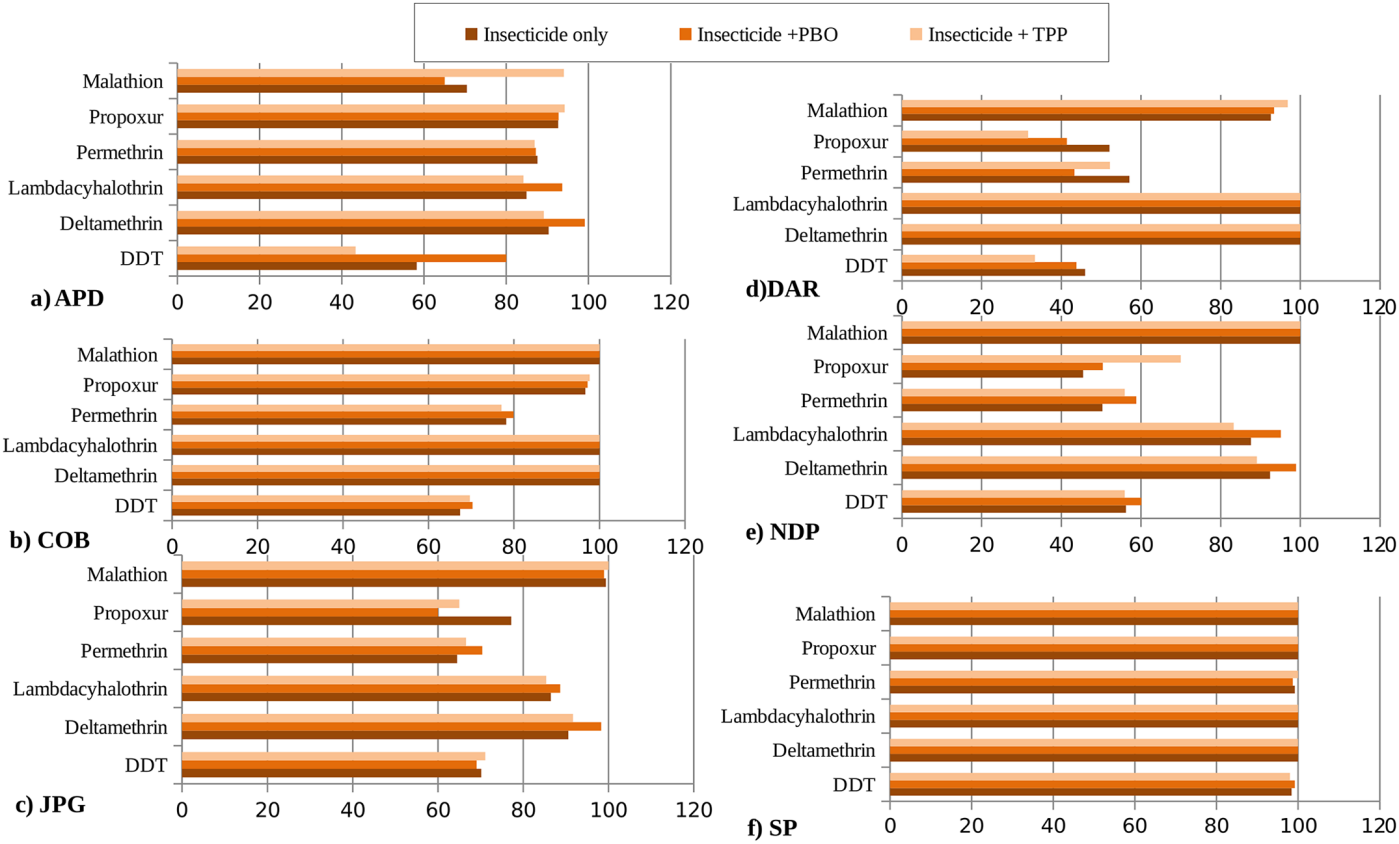
Insecticide susceptibility status against six adulticides among wild *Ae*. *aegypti* populations from northern districts of West Bengal.

The study of adult bioassays revealed that multiple resistance was prevalent among the tested populations against an array of insecticides (Fig 2). All the tested populations were reported to exhibit reduced mortalities against DDT with the highest mortality percentage of 70.2%. Neither CCEs nor CYP_450_s could be assigned as the detoxifying enzyme governing the resistance against DDT, since in only one population (*i*.*e*. APD), PBO exposure was found to enhance susceptibility to DDT whereas in others no such involvement could be noted (Fig 2). Against synthetic pyrethroids, the lowest mortality percentages were recorded against permethrin, *i*.*e*. 50% to 87.6%. Against deltamethrin and lambdacyhalothrin, three of the tested populations *i*.*e*. APD, JPG, NDP possessed unconfirmed/incipient resistance, whereas rest were found to be susceptible. In APD, JPG and NDP population, susceptibility was found to be restored when prior exposure to PBO was done thereby indicating the role of CYP_450_s in resistance against deltamethrin and lambdacyhalothrin. Two out of six tested populations showed mortality percentages below susceptible level against Malathion (Fig 2). In one population, *i*.*e*. APD, use of TPP was found to restore the susceptibility against malathion, enhancing the mortality rate from 70.40% to 94% (S1 Table). Against propoxur, mortality percentages were noted to range from 45.45% to 97.70%, and probable role of CCEs could be assigned to confer such resistance in NDP population as evident through synergism study.

All the tested populations were completely susceptible to both the concentrations of temephos except, one population, *i*.*e*. NDP with mortality percentages 92.5% (0.0200 ppm) and 79.8% (0.0125 ppm) respectively (Table 2). The use of TPP along with temephos could restore the mortality percentage to susceptible levels (Fig 2). All the tested populations exhibited their respective RR50 values ranging from 1.65 to 35.09. The highest RR50 value, *i*.*e*. 35.09 was exhibited by NDP population followed by JPG population with RR50 value of 9.30.

**Table 2:** Susceptibility to temephos in larval *Ae*. *aegypti* collected from districts of northern Bengal.

Sites	Mortality %age (0.0200 ppm)	Mortality %age (0.0125 ppm)	RR50
**APD**	100	100	3.00
**COB**	100	100	1.65
**JPG**	100	100	9.30
**DAR**	100	100	5.43
**NDP**	92.5	79.8	35.09
**SP**	100	100	--

### Knockdown rates

The lowest KDT_95_ value against DDT was noted from SP, *i*.*e*. 121.41, whereas DAR population recorded the highest KDT_95_ value (Table 3). Against, deltamethrin, JPG population recorded the highest KDT_95_ value of 108.21. COB and APD population reported the greatest KDT_95_ value, 32.55 and 76.01 against lambdacyhalothrin and permethrin respectively.

**Table 3:** Knockdown rates (KDT_50_ and KDT_95_) of different *Ae*. *aegypti* populations against tested organochlorine and synthetic pyrethroid insecticides.

Sampling site	DDT		Deltamethrin		Lambdacyhalothrin		Permethrin	
	KDT_50_ _(min)_	KDT_95_ _(min)_	KDT_50_ _(min)_	KDT_95_ _(min)_	KDT_50_ _(min)_	KDT_95_ _(min)_	KDT_50_ _(min)_	KDT_95_ _(min)_
*APD*	142.16	239.31	52.42	101.6	46.91	75.16	37.42	76.01
*COB*	96.34	191.17	14.11	52.19	9.41	32.55	43.66	106.01
*JPG*	95.41	182.35	71.16	108.21	43.33	82.21	52.76	121.32
*DAR*	182.16	272.06	31.40	72.33	12.60	49.36	67.66	161.15
*NDP*	155.39	254.12	58.77	93.9	34.04	45.45	78.98	192.22
*SP*	81.69	121.41	8.69	42.31	9.15	39.18	39.18	78.69

### Biochemical enzyme assay

The activity of major detoxifying enzyme groups were varying among different field caught populations of *Ae*. *aegypti* (Table 4). The activity of α-CCEs ranged from 1.12 to 3.12 times that of the SP, *i*.*e*. 0.241 to 0.668 μmoles mg protein^-1^ min^-1^. Similarly, the activity of β-CCEs among the field populations of *Ae*. *aegypti* ranged from 0.181 to 0.406 μmoles mg protein^-1^ min^-1^. The level of CYP_450_ monoxygenase activity and GST activity were similar throughout the tested mosquito populations, ranging from 0.044 to 0.063 nmoles mg protein^-1^ min^-1^ and 0.32 to 0.42 GSH-CDNB conjugate μM mg protein^-1^ min^-1^ respectively.

**Table 4:** Activities of major detoxifying enzymes in different field caught populations of *Ae*. *Aegypti*.

Sites	α-CCEs (μmoles mg protein^-1^ min^-1^) ± S.E.	β-CCEs (μmoles mg protein^-1^ min^-1^) ± S.E.	CYP_450s_ (nmoles mg protein^-1^ min^-1^) ± S.E.	GSTs (μMmg protein^-1^ min^-1^) ± S.E.
**APD**	0.313 ± 0.008^b^*	0.226 ± 0.008^b^	0.056 ± 0.0002^b^	0.39 ± 0.006^a^
**COB**	0.241 ± 0.004^a^	0.181 ± 0.001^b^	0.044 ± 0.0003^a^	0.32 ± 0.002^a^
**JPG**	0.279 ± 0.001^b^	0.217 ± 0.007^b^	0.063 ± 0.0011^b^	0.42 ± 0.001^a^
**DAR**	0.359 ± 0.007^b^	0.224 ± 0.006^b^	0.059 ± 0.0009^b^	0.41 ± 0.009^a^
**NDP**	0.668 ± 0.021^c^	0.406 ± 0.007^c^	0.057 ± 0.0021^b^	0.33 ± 0.009^a^
**SP**	0.214 ± 0.002^a^	0.161 ± 0.009^a^	0.040 ± 0.0005^a^	0.31 ± 0.002^a^

*Within columns, means followed by the same letter do not differ significantly (P = 0.05) in Tukey’s multiple comparison test (HSDa).

## Discussion

The objective of this study was to reveal the mechanism of prevailing insecticide resistance in the wild populations of *Aedes aegypti* in five dengue endemic districts of sub-Himalayan West Bengal. To determine the underlying mechanisms of insecticide resistance, detoxifying enzymes’ activity, synergism assays and determination of knockdown times, *i*.*e*. KDT_50_ and KDT_95_ were assessed.

In this study, none of the tested *Ae*. *aegypti* populations were found to be susceptible to DDT (Fig 2), with mortality percentages ranging from as low as 46% to 70.2% and 98.4% for SP (Fig 2). The KDT_50_ and KDT_95_ values were also significantly higher than the SP for the field populations of *Ae*. *aegypti* indicating the inefficacy of DDT in dengue vector control. The *Ae*. *albopictus* populations from nearby regions of West Bengal have also been found to possess similar levels of resistance against DDT [18], This result points on the existing DDT selection pressure on *Ae*. *aegypti* and other mosquito vector populations throughout the study region, which may be pertained to the widespread use of DDT in both agriculture and public health sector throughout the world since the past 70 years [1]. DDT resistance have been linked either by sodium channel mutations leading to target site insensitivity [19] or through enhanced detoxification by insecticide detoxifying enzymes, *i*.*e*. GSTs [20], CYP_450_s [1] or CCEs [21]. However, the use of CCE and CYP_450_ inhibitors before DDT exposure showed no significant change in mortality percentage in all populations except APD. The partial recovery of susceptibility to DDT in APD population using PBO, suggests the possible role of CYP_450_s in metabolisation of DDT as in other mosquito vectors such as CYP6M2 gene in *An*. *gambiae* [22]. The involvement of GSTs in the observed resistance against DDT could not be studied, since no significant difference was found in GST activity among the field populations. However, the presence of target site insensitivity, *i*.*e*. kdr mutations needs to be explored to characterize the exact mechanism/s of resistance against DDT [19].

Most of the tested populations were found to be susceptible or incipiently resistant to deltamethrin and lambdacyhalothrin In some of the populations, namely, APD, JPG and NDP the KDT_50_ and KDT_95_ values were also greater implying the onset of resistance against these two pyrethroid insecticides [23]. Since, long lasting insecticide treated nets mainly use deltamethrin as the active insecticide in India [6], the progress of such resistance needs regular monitoring. Against permethrin, varied pattern of resistance was noted ranging from resistant to susceptible levels. In India, synthetic pyrethroids are widely used throughout different agricultural fields to manage the pest populations. The observed resistance against synthetic pyrethroids could be a result of cross exposure or contamination of mosquito habitats by pyrethroids sprayed on agricultural fields [7,9–10]. Against synthetic pyrethroids either metabolic detoxification by CYP_450_s (or other detoxifying enzyme classes) or presence of kdr mutations are known to confer resistance in *Ae*. *aegypti* [24,25–26]. Results of synergism tests revealed that in majority of the populations incipiently resistant to deltamethrin or lambdacyhalothrin, PBO exposure was found to restore (either completely or partially) the susceptibility to these two insecticides, thereby suggesting the role of CYP_450_s behind the altered susceptibility. Moreover, the CYP_450_ activity levels were also significantly higher in APD, JPG and NDP populations, thereby supplementing the results of synergistic study. Many populations of *Ae*. *aegypti* have been found to possess CYP_450_s mediated resistance against deltamethrin or lambdacyhalothrin worldwide [1,26,27]. However, in case of resistance against permethrin, though the detoxifying enzyme activity indicated the possible role of CYP_450_s in JPGand DAR population and CCEs in DAR and NDP population, yet the inefficacy of enzyme inhibitors in enhancing the toxicity of permethrin on any of the field populations of *Ae*. *aegypti* strikes out the involvement of metabolic detoxification behind the observed resistance. Moreover, the similar pattern of resistance against both permethrin and DDT imparts light on the possible role of kdr mutations [19, 25–26] in conferring resistance against both the insecticides having same target site of action. Presence of kdr mutation providing resistance against both pyrethroid and organochlorine insecticides have been found in different mosquito vector species such as *Ae*. *aegypti* [19] and *An. gambiae [28]*. Insecticide susceptibility test against malathion revealed the presence of two resistant or possibly resistant population, *i*.*e*. APD and DAR amongst the six tested populations. Against organophosphate insecticides the prime mechanisms of resistance have been found to be either through enhanced detoxification by enzymes, mainly CCEs [29] or through insensitive AchE [21]. In both APD and DAR populations, pre exposure to CCE inhibitor TPP could moderately enhance the mortality percentages from 70.4% to 94% and 92.6% to 96.8% respectively. Moreover, the significantly higher activities of both α- and β-CCEs also points on the presence of malathion specific CCE mechanism mediated resistance to be prevalent in these populations [30].

Resistance against one more insecticide was tested, *i*.*e*. propoxur, a carbamate insecticide, three field populations (JPG, DAR and NDP) were found to be resistant, whereas remaining (APD and COB) were found to possess unconfirmed resistance against propoxur (Fig 2). Propoxur is not used in India for mosquito control [6], so the presence of propoxur resistant (or incipiently resistant) populations of *Ae*. *aegypti* seems to be a result of accidental exposure to propoxur (via pest control tools) or cross resistance to other xenobiotics [31]. The resistance mechanisms providing resistance against propoxur are generally similar to mechanisms of organophosphate resistance. In one of the tested population, *i*.*e*. NDP resistance against both propoxur and temephos was noted along with an increased activity of CCEs. Furthermore, pre exposure to TPP, was found to restore the susceptibility against both the insectides, implying the possibility that CCEs mediated detoxification may be governing the cross resistance between propoxur and temephos in NDP population.

Majority of the studied larval *Ae*. *aegypti* populations were found to be susceptible to temephos except one population *i*.*e*. NDP population. The NDP population was reported to possess the highest RR50 value, *i*.*e*. 35.09, as well as the lowest mortality percentages among the tested populations (incipient resistance against 0.0200 ppm and resistance against 0.0125 ppm of temephos (Table 2). The NDP mosquito population were collected from areas around the ASEAN trade network highway, the consequences of possessing such insecticide resistance thus appears dangerous to not only India but to neighbouring countries also. The presence of mosquito population resistant to temephos seems to be an obvious result of regular spray of temephos as the choice of larvicide against both dengue and malaria vector control in Governmental and corporate sectors of India [6]. Since, temephos is the widest used larvicide in India [6], development of resistance against this larvicide may have serious implications in dengue prevention efforts [32].

Mainly, the insecticide detoxification enzyme groups associated with resistance against temephos are CCEs [33–34], however some studies also suggest the role of other detoxifying enzymes such as CYP_450_s and GSTs [35]. The use of synergist TPP but not PBO was found to restore the susceptibility to temephos in NDP population, thus pinpointing the mechanism of temephos resistance in this population to be CCE mediated metabolic detoxification. Through the results of detoxifying enzyme activity also, similar inference could be made since the activity of α- and β- CCEs were noted to be significantly higher in NDP population compared to other tested populations (Table 4). Involvement of CCEs in development of resistance against temephos have also been noted in many populations of *Ae*. *aegypti* throughout the world [33–34, 36].

## Conclusion

For an efficient vector control, the instance of insecticide resistance against such a multiple group of insecticides needs proper attention and action. In that context, regular monitoring throughout the study area is inevitable. Furthermore, to gain a complete knowledge of prevailing insecticide resistance mechanism, the mapping of kdr mutations throughout the study region must be done. In some of the *Ae*. *aegypti* populations, where use of synergists along with insecticide could enhance the potency of insecticide must be taken into account when devising an *Aede*s control strategy. From this study, the use of deltamethrin and lambdacyhalothrin seem to be the choice of insecticide for *Ae*. *aegypti* control throughout the study region. Indian Government may introduce newer strategies for integrated vector management such as newer compounds *i*.*e*. etofenprox, neonecotenoids, azadirachtin *etc* or techniques such as or dopamine receptor antagonists as insecticides or introduction of sterile male mosquito seem to hold potential for mosquito control in future. Advanced studies focusing at gene level may also help gain detailed knowledge about the resistance phenomenon.

## Supporting information

S1 Table Insecticide susceptibility status of adult *Ae*. *aegypti* against six adulticides. (DOCX)
