# Supplementary material for: Multiple insecticide resistance mechanisms in primary dengue vector, Aedes aegypti (Linn.) from dengue endemic districts of sub-Himalayan West Bengal, India
Source: PLoS One. 2018 Sep 10;13(9):e0203207. doi: 10.1371/journal.pone.0203207 (PMC6130861; doi:10.1371/journal.pone.0203207)
Supplement: S1 Table — (DOCX) [file pone.0203207.s001.docx]

**S1Table: Insecticide susceptibility status of adult *Ae. aegypti* against six adulticides.**

| **INSECT**  **ICIDES** | **Sampling sites** | | | | | | |
| --- | --- | --- | --- | --- | --- | --- | --- |
|  |  | **APD** | **COB** | **JPG** | **DAR** | **NDP** | **SP** |
|  | **DDT** | 58.2 | 69.7 | 70.2 | 46.0 | 56.2 | 98.4 |
|  | **DDT+PBO** | 80.0 | 67.4 | 69.09 | 43.8 | 60.1 | 99.2 |
|  | **DDT+TPP** | 43.3 | 70.3 | 71.14 | 33.3 | 55.9 | 98.0 |
|  | **Deltamethrin.** | 90.3 | 100.0 | 90.6 | 100.0 | 92.4 | 100.0 |
|  | **Deltamethrin.+PBO** | 99.1 | 100.0 | 98.3 | 100.0 | 98.9 | 100.0 |
|  | **Deltamethrin +TPP** | 89.1 | 100.0 | 92.7 | 100.0 | 89.1 | 100.0 |
|  | **Lambdacyhalothrin** | 84.9 | 100.0 | 86.5 | 100.0 | 87.6 | 100.0 |
|  | **Lambdacyhalothrin+PBO** | 93.6 | 100.0 | 88.7 | 100.0 | 95.1 | 100.0 |
|  | **Lambdacyhalothrin+TPP** | 84.2 | 100.0 | 85.4 | 100.0 | 83.3 | 100.0 |
|  | **Permethrin** | 87.6 | 77.1 | 64.5 | 57.1 | 50.0 | 99.2 |
|  | **Permethrin+PBO** | 87.2 | 78.2 | 69.7 | 43.3 | 58.8 | 98.7 |
|  | **Permethrin+TPP** | 88.9 | 79.9 | 66.6 | 52.7 | 55.9 | 100.0 |
|  | **Malathion** | 70.4 | 100.0 | 99.4 | 92.6 | 100.0 | 100.0 |
|  | **Malathion+PBO** | 65.0 | 100.0 | 99.0 | 93.4 | 100.0 | 100.0 |
|  | **Malathion+TPP** | 94.0 | 100.0 | 100.0 | 96.8 | 100.0 | 100.0 |
|  | **Propoxur** | 92.6 | 97.7 | 77.2 | 52.1 | 45.4 | 100.0 |
|  | **Propoxur+PBO** | 92.7 | 96.7 | 60.0 | 42.3 | 50.3 | 100.0 |
|  | **Propoxur+TPP** | 94.2 | 97.2 | 65.0 | 31.6 | 70.4 | 100.0 |
